# The nexus of social alliances and diverse moral domains: a bedrock for participatory clinical research

**DOI:** 10.3389/fmed.2023.1250247

**Published:** 2023-10-02

**Authors:** Johanna M. C. Blom, Veronica Rivi, Fabio Tascedda, Luca Pani

**Affiliations:** ^1^Department of Biomedical, Metabolic and Neural Sciences, University of Modena and Reggio Emilia, Modena, Italy; ^2^Center for Neuroscience and Neurotechnology, University of Modena and Reggio Emilia, Modena, Italy; ^3^Department of Life Sciences, University of Modena and Reggio Emilia, Modena, Italy; ^4^Consorzio Interuniversitario per le Biotecnologie, Trieste, Italy; ^5^Department of Psychiatry and Behavioral Sciences, University of Miami, Miami, FL, United States

**Keywords:** health data-sharing, clinical trials, decision-making, informed consent, social alliance

## Introduction

Globally, numerous initiatives are converging on the importance of promoting a participatory clinical research approach. The crux of this movement is to empower clinical trial participants with the access and capability to utilize, their health data, predominantly with a focus on enhancing health data sharing. Such endeavors not only foster informed medical decision-making but also catalyze future research pursuits (1). A prime exemplar of these efforts is the Multi-Regional Clinical Trials Center (mrctcenter.org), dedicated to bolstering the integrity, safety, and vigor of clinical trials on an international scale. This organization draws upon the expertise of multidisciplinary teams from diverse sectors—industry, academia, advocacy groups, non-profit organizations, and regulatory agencies—to address pressing issues in clinical trial conduct and oversight (2). In parallel, TransCelerate BioPharma Inc. (www.transceleratebiopharmainc.com), an industry-wide collaboration, seeks to enhance the efficiency, effectiveness, and quality of new drug delivery. It aspires to develop practical resources that empower sponsors to provide access to and facilitate the return of, participants' clinical trial data (3).

Also, the Innovative Health Initiative (IHI) (www.ihi.europa.eu) extends support to public-private partnerships that kindle collaborative projects between industry, academia, patients, regulators, and more. The initiative is designed to transform health research into actionable benefits and advance patient-centric health research across Europe. A core tenet of IHI is to translate health research and innovation into palpable benefits for patients and society, ensuring Europe's ongoing leadership in interdisciplinary, sustainable, and patient-centric health research (4). A recent project launched by IHI, the Framework for Clinical Trial Participants Data Reutilization for a Fully Transparent and Ethical Ecosystem (FACILITATE) (facilitate-project.eu), builds and extends upon previous initiatives. FACILITATE aims to reshape the patient's role in the strategy and design of clinical trials. It empowers patients with new rights and responsibilities, making them active contributors to the drug development process.

While each initiative represents a step toward an ecosystem for data sharing and reuse, combined they converge on the collective objective of crafting data-sharing and re-use protocols that operate within an ethical, legal, and regulatory framework and aim to provide coherent guidelines for all stakeholders, ensuring alignment with the interests of study participants, hospitals, academia, and industry. The ultimate shared success of these initiatives relies on their capacity to build trust by enhancing ethics, safety, and transparency within clinical trials (5). Because participation in clinical trials involves an exchange of personal data for potential health benefits, it is crucial to establish an ethically sound, inclusive decision-making framework that takes these principles into account to avoid potential harm (6). In the present manuscript, we discuss why a social alliance among all study participants should be a primary objective in the clinical research setting. We envision our main aim as fostering a more participatory clinical research paradigm, with the initial focus of the social alliance being on health data sharing and hold a firm conviction that integrating transparency in sharing clinical trial data enhances trust, builds social alliances, and ultimately contributes to shared decision-making processes (7, 8).

## Building on social alliances

The “social alliance”, as we envision it, signifies voluntary collaborations between multiple entities, each possessing distinct structural nuances (9, 10). These entities, encompassing a comprehensive array of stakeholders as highlighted in ln51, pool together their resources, expertise, and capabilities to navigate intricate challenges. The essence of such alliances is rooted in interdependence, a genuine regard for one another's perspectives, unwavering commitment to fortifying the alliance's framework, and an alignment in ethos and values. Importantly, the term “stakeholders” in this context transcends the limited scope of merely “study participants,” encompassing a broader spectrum of individuals and organizations invested in the success of clinical trials. Furthermore, our vision is 2-fold: firstly, a higher-level social alliance that addresses overarching issues affecting all clinical trials, and secondly, a more localized application wherein each clinical trial contextually adopts the principles of the alliance.

The defining features of a social alliance incorporate several elements. First, it necessitates the mutual interdependence of participants and other stakeholders, thereby acknowledging the alignment of their interests and concerns. In this context, the term 'social alliance' refers to the bonds that unite the various stakeholders involved in clinical research and the governance structures that define and sustain these relationships (9, 10). Second, a social alliance embodies an authentic interest in others and their wellbeing, cultivating a sense of connection and mutual trust (7). This involves the recognition that each participant brings a unique perspective and value to the process, thereby enhancing the overall collective experience.

Third, it signifies a commitment to support the structures that allow a social alliance to exist, thereby acknowledging the importance of collective efforts and the power of communal action (11). This can range from shared data management systems to collaborative decision-making processes, all of which can improve the efficiency and effectiveness of the research process.

## Decision-making, morality, and clinical trials

The cognitive mechanics of decision-making incorporate intuitive, emotional, and moral dimensions (12–17). Such dimensions have emerged as critical variables in interpreting the multifaceted social, cultural, ethical, and legal aspects of clinical trial landscapes (18–20). Conventional thinking has commonly emphasized the importance of logical and transparent decision-making processes. However, gaining a thorough understanding of *what* is deemed “reasonable” in a particular situation and/or identifying *who* should be involved in the process requires deeper insight. Individuals around the world face diverse challenges and respond based on their unique cultural backgrounds, which, in turn, guide moral judgments and decision-making processes (21, 22). This leads to an expansive moral domain with several moral foundations, which extend beyond harm and fairness.

As per Haidt's *moral foundation theory* (23–25), six universal foundations shape judgments of morality: Care/harm, Fairness/cheating, Loyalty/betrayal, Authority/subversion, Sanctity/degradation, and Liberty/oppression. These principles are tied closely with specific emotions and virtues, which confer adaptive benefits and contribute to personal success. Relating this to clinical research, we must question: *Which of these principles take precedence?* We think that Principles like Care/harm, Fairness/cheating, and Loyalty/betrayal are of significant relevance. As individuals opt to partake in a clinical trial, they often hope for access to innovative treatments, fueling a sense of care and concern. Yet, this feeling can be overshadowed by fears and potential harm if the treatment outcomes fall short of expectations (26).

## The future of clinical research

The future of participatory clinical research hinges on the ability to cultivate and sustain such social alliances, navigate intricate moral domains, and meet the evolving needs of all stakeholders. Thus, by fostering an environment of integrity, transparency, inclusivity, and reciprocity we can ensure ethical, person-centered clinical research.

Indeed, the integrity of the decision-making process in clinical trials is intrinsically linked to the context in which it occurs. This context is multi-dimensional, comprising scientific, social, ethical, and policy-oriented aspects, further complicated by the interplay between diverse actors. These actors include healthcare professionals, scientists, policymakers, and participants, all of whom are motivated by their values and goals (27).

Although aligning these diverse interests and values is no easy task, the shared goal of advancing medical knowledge and improving human health can serve as a unifying force (28) but may not be enough. We think that by engaging in open dialogues, these shared values can be highlighted, enabling a collective understanding and promoting cooperative actions (29). The emerging paradigm of shared decision-making not only embodies this ethos of collective understanding but also encourages participants to make informed decisions about their health, fostering a sense of autonomy and control (30). Yet, it also acknowledges the interconnectedness of people's lives, emphasizing the need for respectful, considerate actions that do not undermine the wellbeing of others.

As such, shared decision-making becomes an act of moral behavior, contributing to a more equitable, respectful, and compassionate society (31). Contrary to common beliefs, participants are often competent at balancing conflicting values and incorporating both rational assessments and emotional responses into their decision-making processes (32). In other words, when assessing the risks and benefits of trial participation, individuals balance their emotional reactions with analytic thinking. The intricate interaction between emotion and reasoning in clinical trial decision-making is well-emphasized in the *dual-process theory of moral cognition* (33–36), according to which, fast evaluations of the moral significance of potential outcomes enable more elaborate judgments, impacting decisions like whether to participate, for example, in a double-blind placebo control clinical trial where the participant signs an informed consent whereby a 50% probability of not receiving an active treatment is authorized.

A significant challenge, then, lies in mapping the dynamic interplay among values, characteristics, and principles upheld by diverse stakeholders and assessing the quality of relationships between them at different stages of the clinical trial.

If all involved parties, including participants, physicians, and sponsors, agree that decisions are made with accountability and transparency, while recognizing values such as fairness, adherence to standards, and justice, a transparent and trust-filled context can be created, forming the basis of a social alliance (37).

## Conclusions

Biomedical research has experienced significant advancements, yet many challenges remain. Among these is the urgent need for developing and implementing more efficient and ethical strategies for data sharing and participant protection. Meeting these challenges will require continued efforts to understand and address the emotional, and cognitive aspects of decision-making in clinical trials. This requires considering the socio-technological context, the relationships between stakeholders, and the cultural and moral dimensions that shape these relationships (38). In light of the complexities associated with constructing a robust social alliance, we warrant the need for a clear roadmap to guide its formation and progression. At its inception, it is imperative to take deliberate initial steps, grounded in broad consultations and inclusive dialogues. Central to this process is the identification and engagement of key stakeholders, representing a myriad of perspectives, ensuring the alliance is both representative and effective. Additionally, we underscore the importance of establishing dedicated leadership roles within the alliance—individuals or entities who possess the commitment, insight, and authority to steer the alliance toward its ambitious goals. By anchoring this approach in these guiding principles, we aspire to create a social alliance that is not only responsive to current challenges but is also adaptable to the evolving landscape of clinical research.

As we move forward, it's essential to remember that the guiding principles of clinical research, such as respect for persons, beneficence, and justice, remain fundamental (37). These principles anchor us to the purpose of our work, providing a compass to navigate the complexities and challenges that we face. Not least, they remind us that, at its core, clinical research is a deeply human endeavor, rooted in our shared desire to understand, heal, and improve the human condition (39).

## Author contributions

All authors listed have made a substantial, direct, and intellectual contribution to the work and approved it for publication.
